# Directional Slack-Based Measure for the Inverse Data Envelopment Analysis

**DOI:** 10.1155/2014/138923

**Published:** 2014-04-27

**Authors:** Ali Mirsalehy, Mohd Rizam Abu Bakar, Lai Soon Lee, Azmi B. Jaafar, Maryam Heydar

**Affiliations:** ^1^Department of Mathematics, Faculty of Science, Universiti Putra Malaysia, 43400 Serdang, Selangor, Malaysia; ^2^Institute for Mathematical Research, Universiti Putra Malaysia, 43400 Serdang, Selangor, Malaysia; ^3^Faculty of Computer Science and I.T, Universiti Putra Malaysia, 43400 Serdang, Selangor, Malaysia

## Abstract

A novel technique has been introduced in this research which lends its basis to the Directional Slack-Based Measure for the inverse Data Envelopment Analysis. In practice, the current research endeavors to elucidate the inverse directional slack-based measure model within a new production possibility set. On one occasion, there is a modification imposed on the output (input) quantities of an efficient decision making unit. In detail, the efficient decision making unit in this method was omitted from the present production possibility set but substituted by the considered efficient decision making unit while its input and output quantities were subsequently modified. The efficiency score of the entire DMUs will be retained in this approach. Also, there would be an improvement in the efficiency score. The proposed approach was investigated in this study with reference to a resource allocation problem. It is possible to simultaneously consider any upsurges (declines) of certain outputs associated with the efficient decision making unit. The significance of the represented model is accentuated by presenting numerical examples.

## 1. Introduction

Data envelopment analysis (DEA) is a nonparametric linear programming-based technique for measuring and evaluating the relative performances of a set of decision making units (DMUs). Each of the DMUs uses multiple inputs to generate multiple outputs and they are assumed to operate under similar conditions. By considering the information about available data on the input/output values of the DMUs and some axiomatic foundations, a production possibility set (PPS) is defined. The basic ideas behind the DEA date back to Farrell [1], but the recent series of discussions started with study conducted by Charnes et al. [2], in assessment of an educational center in the USA and extended by Banker et al. [3]. Many researchers have proposed extensions to the DEA technique (see Emrouznejad et al. [4] and Gattoufi et al. [5]).

If it is intended to measure the efficiency, the DEA models can be generally categorized into two main groups, namely, the radial and nonradial models. Farrell [1] and Debreu [6] were pioneers to conduct the first systematic surveys on the radial models. Seemingly, the radial-measures approach entails various striking characteristics. Yet, it suffers from various problems in use notwithstanding the intensity of this approach (see Avkiran et al. [7]). Moreover, Koopmans [8] and Russell [9] conducted some of the earliest efforts on the nonradial models. For the time being, various undertakings were exerted on the basis of the performance approximation with the aim of elucidating the nonradial measure for the technical efficiency (see Charnes et al. [10] and Cooper et al. [11–13]), each of which are concomitant with relevant benefits and drawbacks (see Avkiran et al. [7]). In order to acquire a nonradial measure termed the slacks-based measure (SBM) wherein there is a possibility of diminishing the input and output slacks, a newfangled and proper synthetic method was employed by Tone [14] in a controlled study. The Russell measure model was presented in the proposed model of Tone [14] by Färe and Lovell [15] which was additionally reexamined by Pastor et al. [16] introducing it as the Enhanced Russell Measure model (ERM). In recent times, the Directional Slack-Based Measures (DSBM) of efficiency was introduced by Jahanshahloo et al. [17] which worked under the Generalized Returns to Scale (GRS). On the basis of the directional distance function, the GRS is reported to encompass numerous remarkable characteristics.

The point of fact is that the DMUs performances are appraised by the DEA; in this context, the production relationship can be examined devoid of any functional specification between them. We should assume a production technology wherein m inputs are required for engendering the s outputs. In turn, we need to represent the inputs and outputs by *x* and *y*. We can afterward define the production possibility set (PPS) *T*; in this fashion: *T* = {(*x*, *y*) : *y* can be engendered by *x*}. *T* contains the entire possible input/output combinations. Besides, the boundary points of *T* are titled as the efficient frontier, the frontier line, or the production frontier [1]. It needs to be pinpointed that those DMUs which are the properties of this frontier will be considered efficient whereas the others would be regarded as inefficient. The values of the observations' inputs and outputs in the dataset will influentially affect the efficient frontier representation in the DEA. Any relative variations in the input and output amounts will result in the variations in the efficient frontier structure as well as the DMUs' relative efficiency values. At this point, it is necessary to know how not to deteriorate the relative efficiency value of a deliberated DMU in case the internal technical structure of the deliberated DMU marginally fluctuates in a short run.

Some researchers have severely debated the inverse DEA models through the last two decades. Because of the fact that the constraint parameters include the input and output values of the DMUs for the DEA models, the inverse DEA models can be categorized into two kinds. This classification depends on the kind of parameters which are varying and the ones which have to be changed in order for the optimal objective value to be kept unaffected. A resource allocation problem is underscored to be the first category of the inverse DEA models which is known as an inverse DEA problem associated with specifying the utmost probable inputs for certain outputs with the intention of keeping the current efficiency value of a deliberated DMU unvaried in comparison with the other DMUs. Contrariwise, the other inverse DEA model is said to be an investment analysis problem which is an inverse DEA problem associated with identifying the best possible outputs for certain inputs in order to keep the current efficiency value of a certain DMU unchanged as compared with the remainder of DMUs. Ordinarily, there should not be a drastic change in a DMU's internal technical structure in a short run (Yan et al. [18]). The inverse DEA was initially proposed by Wei et al. [19] in recent times with the aim of addressing this question: if given inputs (outputs) of a certain unit are elevated amid a set of DMUs, how much rise should be given to the outputs (inputs) of that same unit while the DMU maintains its efficiency level in comparison with the other DMUs?

Alternatively, an inverse measure was defined by Cherchye and van Puyenbroeck [20] on the basis of the efficient reference mix properties. A method was also introduced by Pendharkar [21] in which the DEA was being utilized to resolve the inverse classification problem. Such a problem indeed implicates discovering the way predictor attributes of a case are changed so that such a case would be categorized into a diverse and more appropriate category. This study revealed that the DEA might be exploited for the inverse classification problem assuming the classes' conditional monotonicity and convexity. The inversed DEA problem along with the developed solution method proposed by Yan et al. [18] was extended by Jahanshahloo et al. [22] to the case of specifying the outputs of a certain DMU once the entire or a few of the inputs had a rise as well as the time when the efficiency value of such DMU, as compared with the other DMUs, was required to be enhanced by its current efficiency value identified percentage. Through another study executed by Jahanshahloo et al. [23], the inversed DEA models were found to be utilized for calculating the inputs for a DMU once the entire or a few of the outputs as well as the DMU's efficiency value were either elevated or retained. They likewise acknowledged the extra inputs once the outputs could be calculated by making use of the models recommended by Yan et al. [18] and Jahanshahloo et al. [22].

In line with this, a modified inverse DEA model was introduced by Jahanshahloo et al. [24] intended for the sensitivity analysis of both efficient and inefficient DMUs' efficiency categorizations. In this model, the preference cones were used to represent the essential policies over inputs, outputs, and DMUs. Furthermore, a broader case of the aforementioned question was addressed by Hadi-Vencheh and Foroughi [25]. Their study demonstrated that, in the paper of Wei et al. [19], only the upsurge of inputs (outputs) had been taken in to account while a unit might contend with the upsurge of some of inputs (outputs) and the declines of the other inputs (outputs) at the same time. Besides, a method was introduced by Alinezhad et al. [26] which exploited an interactive MOLP aimed at resolving the inverse DEA problems. Nonetheless, it needs to be accentuated that the majority of studies elaborating on the inverse DEA problems have merely concentrated on the considered DMU's efficiency score irrespective of considering the effects of the input (output) changes on the other DMUs' efficiency scores. In recent times, the inverse BCC model was considered by Lertworasirikul et al. [27] for a resource allocation problem in which it was possible to instantaneously deliberate some outputs' upsurges and the other outputs' decreases in the given DMU.

The current research is an attempt to introduce a new inverse DEA model while lending its basis to the efficiency DSBM [17]. So far, this has been discarded by the preceding studies although there have been various methods on the term inverse DEA model. With the intention of enhancing the efficiency scores of some DMUs when there are changes in the output (input) amounts of an efficient DMU, the current study will elucidate the inverse DEA model for the DSBM model. The inverse DSBM model is meant for determining the utmost possible input (output) quantities of the efficient DMU once its outputs (input) are changed. In detail, the deliberated efficient DMU was eliminated from the current PPS, being substituted by the same efficient DMU following the modification of its inputs and outputs. It is asserted here that the introduced inverse DSBM model seemingly is a kind of resource allocation problem in which it is possible to concurrently consider some inputs' rises (reductions). In practice, the mentioned inverse DEA model is advantageous for the decision makers enabling them to realize they way of allocating the limited resources to an efficient DMU the moment its inputs and outputs are modified consistent with the anticipated trivial upsurges of its outputs (inputs). In this way, the modifications would result in a boost in some DMUs' efficiency scores.

The current paper is organized as follows.  Section 2 succinctly introduces the directional distance function and further elaborates on the DSBM model detailing its properties.  Section 3 deals with our proposed model for the invers DEA model, exploring it meticulously to determine the best possible inputs for the intended efficient DMU with the purpose of enhancing the efficiency scores of some of the DMUs. Moreover, Section 4 presents a solution approach to the inverse proposed approach while Section 5 provides the illustrative examples for the invers DEA model. At the end, Section 6 concludes the research as well as presenting some further research.

## 2. Directional Slack-Based Measure

Directional distance function is concisely presented in this section along with describing the DSBM model. Furthermore, its properties as recommended by Jahanshahloo et al. [17] have been also underlined.

Suppose there are *n*  DMU_*j*_ and that the DMU_*j*_ (*j* = 1,…, *n*) are under assessment such that they convert *m* inputs *x*_*ij*_ to *s* outputs *y*_*kj*_ where *x*_*ij*_ ≥ 0, *x*_*ij*_ ≠ 0, *y*_*kj*_ ≥ 0, and *y*_*kj*_ ≠ 0 (*i* = 1,…, *m*, *k* = 1,…, *s*). Indeed, let the *o*th DMU consumes inputs *x*_*io*_ = (*x*_1*o*_, *x*_2*o*_,…, *x*_*mo*_) to generate outputs *y*_*ko*_ = (*y*_1*o*_, *y*_2*o*_,…, *y*_*so*_) which  *x*_*io*_ ≥ 0, *x*_*io*_ ≠ 0, *y*_*ko*_ ≥ 0, and *y*_*ko*_ ≠ 0 (*i* = 1,…, *m*, *k* = 1,…, *s*) and also the PPS is assigned by *T*.

Chambers et al. [28, 29] were pioneers in examining the structural behavior of the directional distance function by which a value is specified for the technical efficiency. The directional distance function is potent of simultaneously heightening the outputs as well as declining the inputs; in other words, the given vector (*x*, *y*) as the input-output vector is reflected onto the PPS production frontier. This can be achieved through utilizing a predetermined direction vector (−*g* ^−^, *g* ^+^) ∈ (−(*R*_+_^*m*^), *R*_+_^*s*^) shown in this fashion:

(1) $$\begin{array}{c} {\vec{D}}_{T}=\left(x,y;-g^{-},g^{+}\right)=\mathrm{Max}\left\{\theta :\left(x-\theta g^{-},y+\theta g^{+}\right)\in T\right\}. \end{array}$$

We can indicate the PPS presented consistent with the standard assumptions by the observed DMUs with general returns to scale (GRS) assumption of technology (Cooper et al. [30]) as

(2) TG={(x,y):x≥∑j=1nxijλj,y≤∑j=1nykjλj,    U≤∑j=1nλj≤L,  λj≥0,  i=1,…,m,   k=1,…,s,  j=1,…,n}

in which two nonnegative scalar parameters exist which include *L* (0 ≤ *L* ≤ 1) and *U* (≥1) for ∑_*j*=1_^*n*^ *λ*_*j*_.

Taking into account *L* = 1 and *U* = 1, the *T*_*G*_ can be altered to *T*_*V*_ which stands as the PPS pertinent variable returns to scale (VRS) assumption of technology (Banker et al. [3]). By bearing in mind *L* = 1, *U* = *∞*, and *L* = 0, *U* = 1, *T*_*G*_ converts into *T*_*ID*_ and *T*_*ND*_ that are referred to as PPS, respectively, pertinent to the increasing returns to scale (IRS) and decreasing returns to scale (DRS) assumption of technology. The reason why the IRS and DRS are recognized as the ‘‘variable returns to scale” is that the engendered outputs have a rise more or a smaller amount, corresponding to the rises in the inputs [31]. Besides, if we assume that *L* = 0 and *U* = *∞*, the *T*_*G*_ changes into *T*_*C*_ which denotes the PPS with respect to the constant returns to scale (CRS) supposition of technology (Charnes et al. [2]).

It needs to be pinpointed that the radial models are unable to explain the nonradial excesses and shortfalls because of the fact that such models remove the nonzero input and output slacks. Relevant to this, the DSBM model of efficiency was introduced by Jahanshahloo et al. [17] in regard to *T*_*G*_ as shown in

(3) Model(1)  ρ∗=Min⁡ 1−(1/m)∑i=1mθi1+(1/s)∑k=1sφk,

(4) s.t.  ∑j=1nλjxij≤xio−θigi−, i=1,…,m,

(5)  ∑j=1nλjykj≥yko+gk+φk, k=1,…,s,

(6)  L≤∑j=1nλj≤U,

(7)  λj≥0, j=1,…,n,

(8)  θi≥0, i=1,…,m,

(9)  φk≥0, k=1,…,s.

Now, by selecting the direction vector *g* = (−*g* ^−^, *g* ^+^) as *g*_*i*_^−^ = *x*_*io*_ (*i* = 1,…, *m*), *g*_*k*_^+^ = *y*_*ko*_ (*k* = 1,…, *s*), we consider the DSBM model by the following model which is a special case of the SBM [14] and the ERM [16] with respect to *T*_*G*_:

(10) Model(2)  ρ∗=Min⁡ 1−(1/m)∑i=1mθi1+(1/s)∑k=1sφks.t. ∑j=1nλjxij≤xio(1−θi),i=1,…,m, ∑j=1nλjykj≥yko(1+φk), k=1,…,s, L≤∑j=1nλj≤U, λj≥0, j=1,…,n, θi≥0, i=1,…,m, φk≥0, k=1,…,s,

while *θ*_*i*_ stands for the contraction rate related to input *i* and *φ*_*k*_ demonstrates the extension rate of output *k* for the *o*th DMU. In this way, it can be reflected onto the production frontier of *T*_*G*_. Likewise, the objective function of the DSBM model is responsible for increasing *θ*_*i*_ and *φ*_*k*_ (*i* = 1,…, *m*, *k* = 1,…, *s*). The subsequent variables *λ*_*j*_ (*j* = 1,…, *n*) will be utilized for a structural connection that is established between the DMUs within the input/output space; that is to say that *λ* ∈ *R* ^*n*^ is the intensity vector.

**Definition 1**

A DMU_*j*_ (*j* = 1,…, *n*) will be efficient if and only if ρ* = 1.

In the optimum solutions, the mentioned situation will be equal to  *θ*_i_* = 0, (*i* = 1,…, *m*) and  *φ*_k_* = 0, (*k* = 1,…, *s*).

**Proposition 2**

Consider 0 ≤ ρ* ≤ 1.

**Proof**

Primarily, because *x*_*io*_(1 − *θ*_*i*_) ≥ 0, ρ* ≤ 1. Alternatively, it is quite known that ρ* ≥ 0. Therefore, 0 ≤ ρ* ≤ 1.

According to Proposition 2, ρ* is taken to mean as an efficiency score; in other words, there is no input inefficiency (excess) and no output inefficiency (shortage) in any optimal solution within the entire inputs and outputs.

**Proposition 3**

The entire input and output constraints in Model (2) are binding in each optimum solution (5).

**Proof**

The proof for this proposition resembles that of Pastor et al. [16].

It is necessary to highlight that the DSBM model entails several favorable confidants which can be inferred as follows.

- The mentioned measure is invariant regarding the measurement unit of every input and output item.
- The measure is observed to be monotone intensely while it is capable of diminishing in each *θ*_*i*_ and *φ*_*k*_.
- The Charnes-Cooper conversions can be exploited for easily transforming this model into a linear programming problem [32].
- It can be said that this measure seems to be complete due to the fact that it is in contradiction with the oriented measures; in practice, the entire inefficiencies which are concomitant with the nonzero slacks which might be recognized by the model are referred to as nonoriented measures.

## 3. The Inverse DSBM Model

In the current study, Model (2) was adjusted through the method mentioned below for our target. Indeed, we replaced the objective function by its numerator while we discarded the denominator. Consequently, the input-oriented Model (2) can be presented in the following way:

(11) Model(3)  ψ∗=Min⁡ 1−1m∑i=1mθi,s.t.  ∑j=1nλjxij≤xio(1−θi), i=1,…,m, ∑j=1nλjykj≥yko, k=1,…,s, L≤∑j=1nλj≤U, λj≥0, j=1,…,n, θi≥0, i=1,…,m.

Seemingly, *θ*_*i*_ (*i* = 1,…, *m*) is the contraction rate in input *i* for the *o*th DMU that might be reflected onto the production frontier of *T*_*G*_. Likewise, ψ* signifies the DMU_*j*_'s efficiency score.

It is to be stated that the linear combination of the observed DMUs failed to cover the efficient frontier related to Model (3); henceforth, the efficient frontier will be moved by every alteration applied to the inputs or outputs of the given efficient DMUs which can put on the efficient frontier. In addition, there might be alterations in the DMUs' efficiency scores in the PPS. This part was started out aimed at recommending an inverse model through making use of Model (3) in order to attain the efficiency scores of every DMU in comparison with the other DMUs on the basis of the acquired efficient frontier. Correspondingly, there might be some improvement in the efficiency scores associated with few of the DMUs. Having regarded the acquired efficient frontier, the PPS would integrate the entire observed DMUs with the objective of removing the given efficient DMU from the present PPS while being substituted by the same efficient DMU following the change in the quantities of its input and output.

We are now enabled to examine Model (3) for the inverse DEA models by being consistent with whatever stated formerly as well as considering the DMUs, as well as their consistent input and output vectors. In addition, DMU_*e*_ can indicate the given efficient DMU with the input and output quantities in progress. Also, the DMU_*e*′_ can be deliberated as an efficient DMU once there is an alteration in its inputs and outputs. At last, the advanced inverse DEA model is introduced for a resource allocation problem while it endeavors to tackle the following problem.

The assumption is that a group of DMUs in progress exists. Then *θ*_*i*_^j*^ (*i* = 1,…, *m*, *j* = 1,…, *n*) indicates the decline rate in *i*th input of DMU_*j*_ from Model (3). Moreover, the output quantities of DMU_*e*_ have experienced an improvement from *Y*_*e*_ to *Y*_*e*_ + Δ*Y*_*e*_ ≥ 0 (Δ*Y*_*e*_ ≠ 0). At this point, the minimum *X*_*e*_ + Δ*X*_*e*_ ≥ 0 (Δ*X*_*e*_ ≠ 0) should be obtained somehow the efficiency score of DMU_*e*_ with new input and output amounts (*X*_*e*_ + Δ*X*_*e*_, *Y*_*e*_ + Δ*Y*_*e*_) remains stable whereas the other DMUs fail to worsen.

The point to be accentuated is that, prior to the modifications of the DMU_*e*_ inputs and outputs, the present PPS was integrated with *n*  DMU_*j*_ (*j* = 1,…, *n*). On the other hand, the new PPS was also integrated with *n*  DMU_*j*_ (*j* = 1,…, *e* − 1, *e*′, *e* + 1,…, *n*) following the modifications of the DMU_*e*_ inputs and outputs. Indeed, DMU_*e*_ was omitted from the present PPS while DMU_*e*′_ was substituted.

In order to conduct the analyses, initially the efficiency score of DMU_*j*_ was acquired prior to the modifications of the DMU_*e*_ inputs and outputs. This was fulfilled by utilizing Model (3) in which *θ*_*i*_^j*^ (*i* = 1,…, *m*, *j* = 1,…, *n*) indicated the decline rate in the *i*th input of DMU_*j*_ prior to the modifications of the DMU_*e*_ output amounts. In addition, *ψ*_j_* signified the DMU_*j*_ efficiency score.

What follows is inverse Model (3) recommended for a resource allocation problem.

**Step 1**

Let us assume that the DMU_*e*_ outputs are transformed from *Y*_*e*_ to *Y*_*e*_ + Δ*Y*_*e*_ ≥ 0 (Δ*Y*_*e*_ ≠ 0). Thus, the minimum *X*_*e*_ + Δ*X*_*e*_ ≥ 0, (Δ*X*_*e*_ ≠ 0) was obtained through the succeeding model:

(12) Model(4)  Min⁡ Xe+ΔXe,

(13) s.t. ∑j=1j≠enλjxij≤(xie+Δxie)(1−θie∗),i=1,…,m,

(14)  ∑j=1j≠enλjykj≥(yre+Δyre), k=1,…,s,

(15)  L≤∑j=1j≠enλj≤U,

(16)  λj≥0, j≠e,  j=1,…,n.

**Step 2**

Δ*X*_*e*_ was used from Model (4) while the efficiency score could be gained by the subsequent model for the other DMU_*p*_ (*p* = 1,…, *n*, *p* ≠ *e*):

(17) Model(5)  γp∗=Min⁡ 1−1m∑i=1mθip,s.t. ∑j=1j≠enλjxij+λe′(xie+Δxie) ≤xip(1−θip), i=1,…,m,∑j=1j≠enλjykj+λe′(yre+Δyre)≥ykp,k=1,…,s, L≤∑j=1j≠enλj+λe′≤U, λj≥0, j≠e, j=1,…,n, λe′≥0.

The *θ*_*i*_^*p*^ (*i* = 1,…, *m*) signifies the decline rate in the *i*th input of DMU_*p*_ (*p* = 1,…, *n*, *p* ≠ *e*) following the implementation of modifications in the output values of DMU_*e*_. Besides, *γ*_p_* demonstrated the efficiency score of DMU_*p*_ (*p* = 1,…, *n*, *p* ≠ *e*).

It needs to be affirmed that Models (4) and (5) were as multiobjective nonlinear programming (MONLP) forms.

## 4. The Solution Related to the MONLP

The Δ*X*_*e*_ value was required to be determined in this section as the aim was to solve the inverse DSBM model for the resource allocation problem such that the efficiency score did not decline for the whole DMUs. For fulfilling this aim, Models (4) and (5) were needed to be solved. Nevertheless, it is admitted that solving these models is not such an easy task because they stand in the form of MONLP. Subsequently, a linear programming model was assumed in Proposition 6, yielding an optimum solution for the inverse model.

**Proposition 4**

The assumption that the efficiency score of *DMU*_*e*_ in connection with other *DMU*_*j*_ in a set of homogeneous *DMU*_*j*_ (*j* = 1,…, *n*) is *ψ*_e_* and *θ*_*i*_^e*^ (*i* = 1,…, *m*) indicates the decline rate in the *i*th input of *DMU*_*e*_. By applying the modifications to the output values of *DMU*_*e*_, Δ*Y*_*e*_ ≠ 0, the minimum Δ*X*_*e*_ of the *DMU*_*e*′_, which fails to worsen the efficiency score of the entire *DMU*_*j*_ (*j* = 1,…, *n*), can be attained through solving Model (4).

**Proof**

*θ*_*i*_^e*^ = 0 (*i* = 1,…, *m*) because DMU_*e*_ is being regarded as an efficient DMU. Consequently, after applying the modifications to the output values of DMU_*e*_, Δ*Y*_*e*_ ≠ 0, the minimum Δ*X*_*e*_ of the DMU_*e*′_ will be acquired through solving Model (4) while it fails to worsen the efficiency score of DMU_*e*′_. Furthermore, the optimal value of Model (5) cannot be less than that of Model (3). Because Model (3) contains feasible regions which are in turn subspaces of the feasible regions belonging to Model (5), *θ*_*i*_^*p*^ ≤ *θ*_*i*_^p*^and 1 − *θ*_*i*_^*p*^ ≥ 1 − *θ*_*i*_^p*^ (*i* = 1,…, *m*). Consequently, for other DMU_*p*_ (*p* = 1,…, *n*,  *p* ≠ *e*), the efficiency scores could be acquired by Model (5) and we would then have 1 − (1/*m*)∑_*i*=1_^*m*^ *θ*_*i*_^*p*^ ≤ 1 − (1/*m*)∑_*i*=1_^*m*^ *θ*_*i*_^p*^; then, *γ*_p_* ≤ *ψ*_p_*.

It is denoted by Proposition 5 that once we execute Steps 1 and 2, the efficiency scores related to the entire DMU_*j*_ (*j* = 1,…, *n*) would not get worse as compared with the efficiency scores related to Model (3). To sum up, by executing the two mentioned steps, it is observed that the efficiency scores associated with all DMU_*j*_ (*j* = 1,…, *n*) would either remain unchanged or experience a rise.

**Proposition 5**

If one assumes that the DMU_*e*_ efficiency score in connection with other DMU_*j*_ in a set of homogeneous DMU_*j*_ (*j* = 1,…, *n*) is *ψ*_e_* and  *θ*_*i*_^e*^ (*i* = 1,…, *m*) demonstrates the decline rate in the *i*th input of DMU_*e*_, besides, we need to assume that the DMU_*e*_ outputs are changed from *Y*_*e*_ to *Y*_*e*_ + Δ*Y*_*e*_ ≥ 0, (Δ*Y*_*e*_ ≠ 0), then, there would be one optimal solution as a minimum to Model (4) if and only if  *Y*_*e*_ + Δ*Y*_*e*_ ∈ *P*_*out*_, where

(18) Pout={y;∑j=1j≠enλjykj≥y(k=1,…,s),   L≤∑j=1j≠enλj≤U, λj≥0(j=1,…,n,j≠e)}.

**Proof**

If *Y*_*e*_ + Δ*Y*_*e*_ ∈ *P*_out_, the constraints will be therefore met in Model (4):

(19) $$\begin{array}{c} \overset{n}{\underset{\begin{array}{c} j=1\\ j\neq e \end{array}}{\sum }}\lambda_{j}y_{kj}\geq \left(y_{re}+\Delta y_{re}\right),\;k=1,\ldots ,s.\\ L\leq \overset{n}{\underset{\begin{array}{c} j=1\\ j\neq e \end{array}}{\sum }}\lambda_{j}\leq U,\\ \lambda_{j}\geq 0,\;j\neq e,\;j=1,\ldots ,n. \end{array}$$

Additionally, having estimated the proper bottommost quantity *X*_*e*_ + Δ*X*_*e*_ ≥ 0 (Δ*X*_*e*_ ≠ 0), the succeeding constraints would be met:

(20) $$\begin{array}{c} \overset{n}{\underset{\begin{array}{c} j=1\\ j\neq e \end{array}}{\sum }}\lambda_{j}x_{ij}\leq \left(x_{ie}+\Delta x_{ie}\right)\left(1-\;\theta_{i}^{e^{*}}\right),\;i=1,\ldots ,m. \end{array}$$

For that reason, there would be one optimal solution to Model (4) as a minimum which will be attained by allowing the objective associated with Model (3). Nonetheless, on the condition that there is one optimal solution as a minimum to Model (4), *Y*_*e*_ + Δ*Y*_*e*_ ∈ *P*_out_  which is consistent with the constraints (14) in Model (4).

**Proposition 6**

Having considered the ensuing linear programming model in which *θ*_*i*_^e*^stands for the decline rate in the *i*th input of DMU_*e*_ in Model (3) and *W* ^*T*^ ∈ *R* ^*m*^, every optimal solution related to this model would be then a Pareto solution for Model (4):

(21) Model(6)  Min⁡  WT(Xe+ΔXe)s.t. ∑j=1j≠enλjxij≤(xie+Δxie)(1−θie∗),i=1,…,m, ∑j=1j≠enλjykj≥(yre+Δyre), k=1,…,s, L≤∑j=1j≠enλj≤U, λj≥0, j≠e,  j=1,…,n.

**Proof**

If we consider (λ*, Δ*X*_e_*) as an optimal solution for Model (6), we need to assume by contrast where it cannot be a Pareto solution to Model (4). Moreover, we have (*λ*, Δ*X*_*e*_) to the Model (4) so that Δ*X*_*e*_ ≤ Δ*X*_e_* without generality loss while as a minimum a severe inequity would be met in any of the components. Consequently, there would exist *W* ^*T*^(*X*_*e*_ + Δ*X*_*e*_  ) ≤ *W* ^*T*^(*X*_*e*_ + Δ*X*_e_*  ), *W* ^*T*^ > 0. Hereafter, it is noticeable that (*λ*, Δ*X*_*e*_) can be taken as the solution for Model (4). Indeed, this should be introduced as an inconsistency and the relevant proof would be subsequently established due to the fact that the sets of constraint resembled in Model (4) and (6).

## 5. Illustrative Examples

Our proposed technique is exhibited here by two simple numerical examples along with one applied example. The models introduced earlier will be utilized for the case of *L* = 1 and *U* = 1 in this section because it seems to be more plenary as compared with the other cases. Also, the GAMS recognized as an operative software package was employed for solving the mentioned models.

**Example 1**

We primarily assume that there are 8 DMUs that make use of one input for producing one output. Then, it would be initially necessary to solve Model (3) if it is intended to attain the DMUs efficiency scores entirely prior to any modifications in the inputs and outputs of the given efficient DMU.  Table 1 represents all the data and results obtained for Model (3) indicating that technically DMUs A, B, D, F, and H appear to be efficient whereas DMUs C, E, and G seem to be inefficient.

At this point, we need to take into account efficient DMU B and to consider that *W* ^*T*^ = (1, 1) for the input weights as well as the DMU B's output is changed from 11 to 10.80. As predicted, we realized that there were no feasible solutions for Model (6) for DMU B once we solved it. In other words, it can be declared that the new output for DMU B did not appear in *P*_out_. Once more, we need to take into account DMU B while assuming a reduction in its output from 11 to 9.80. Subsequently, an optimal solution will be gained as Δ*x* = −2.10. It means that the new output appears in *P*_out_ once Model (6) is solved for it. At this point, the DMUs' efficiency scores can be estimated by Model (5) through utilizing Δ*x* from Model (6) for the other DMUs. Henceforth, it is claimed that that they do not undergo any declines as they stay unaffected. In other words, they do not get worse.

At this instant, another efficient DMU is taken into account while allowing for DMU A. Then, it is necessary to suppose that there would be a rise in its output from 8 to 9. Afterward, one optimal solution will be obtained as Δ*x* = 0.65 once Model (6) is solved for it. In other words, the new output will appear in *P*_out_. All the DMUs' efficiency scores can be estimated using Model (5) by making use of Δ*x* from Model (6) for the other DMUs. Seemingly, it is discerned that the efficiency scores related to DMU C and E underwent a boost from 0.67 and 0.56 to 0.72 and 0.58, respectively. Also, the efficiency scores related to the other DMUs would stay unaffected.

**Example 2**

To better understand the proposed method, we also exploited a two-input one-output five-DMU example. Primarily, Model (3) was solved with the aim of estimating the DMUs' efficiency scores prior to modifications applied to the inputs and outputs of the given efficient DMU.  Table 2 illustrates all the relevant data and results attained for Model (3). Having a glimpse over the efficiency analysis of DMUs reveals that although technically DMUs 3 and 4 seem to be inefficient, DMUs 1, 2, and 5 prove to be efficient.

In case we suppose the efficient DMU 2 and that *W* ^*T*^ = (1, 1) as well as assuming that the DMU 2 outputs were changed from (100, 3) to (93, 6). Then the optimal objective quantities would be equal to 26.70 while the optimal solution would be Δ*x*_1_ = 0.90, Δ*x*_2_ = −4.20 if we solve Model (6) for it. This implies that the new outputs appear in *P*_out_. At this point, we utilized the optimal solution quantities from Model (6) but the DMUs' efficiency scores were all required to be estimated by Model (5) for the other DMUs. Additionally, it was observed that the efficiency scores associated with DMU 4 experienced an enhancement from 0.86 to 1. Furthermore, there was no change in the other DMUs' efficiency scores. It can be pinpointed that they did not get worse. It can be asserted hereby that no feasible solution will exist for Model (6) on the condition that the DMU 2 outputs are converted from (100, 3) to (95, 6), for example.

**Example 3**

We aim at examining the methods more applicably to expose their competencies; we are then urged to presume an empirical example on the die press division of a motorcycle-part company [27]. In detail, the motorcycle parts are formed by the die press division using the die press machines. In the die press division, there exist a set of 25 die press machines having 80-ton press. In the current research, the die press machines have been assumed as the DMUs. To be more precise, Table 3 indicates that the mentioned company used 9 variables from the dataset having 6 inputs and 4 outputs characterized as *x*_1_, *x*_2_, *x*_3_, *x*_4_, *x*_5_, and *x*_6_ for the inputs as well as *y*_1_, *y*_2_, and *y*_3_ for the outputs. It needs to be emphasized that the 7th input was eliminated from the dataset. Table 3 demonstrates the data and results obtained for Model (3) on the efficiency analysis, indicating that only 7 technically inefficient DMUs exist including DMU 4, DMU 9, DMU 10, DMU 15, DMU 20, DMU 23, and DMU 24 whereas the other DMUs seem to be entirely efficient technically.

Now, we take into account efficient DMU 3, assuming that the output vector related to DMU 3 has been converted from (906, 1.29, 96) to (861, 1.32, 99) as well as assuming that *W* ^*T*^ = (1, 1, 1, 1, 1, 1) for the input weights. Then it was revealed that no feasible solutions existed to this problem after having solved Model (3) for DMU 3. The results here resembled that of proposition 4 showing that the DMU 3 new output values were not in *P*_out_. Consequently, the new input vector in this case was not detected to retain or enhance the efficiency sores related to some of the DMUs if the DMU 3 outputs are converted to (861, 1.32, 99). Again, we take into account DMU 3 while it is assumed that the DMU 3 output vector is transformed from (906, 1.29, 96) to (861, 1.30, 99). Seemingly, the new output amounts appear in *P*_out_. Having solved Model (3) for DMU 3, it is observed that the optimal solution would be Δ*x*_1_ = 3.28, Δ*x*_2_ = −0.09, Δ*x*_3_ = −4.19, Δ*x*_4_ = 0.94, Δ*x*_5_ = 5, Δ*x*_6_ = 39.06 while the optimal objective amount would be 337. Consequently, it is observed that the new input vector would be (21.28, 6.91, 78.81, 110.94, 10, 109.06). Moreover, Model (5) is employed for estimating the efficiency score of the entire DMUs utilizing the new input and output vectors for DMU 3 for the other DMUs. Here, it is discerned that the efficiency scores associated with DMU 20 and 24 underwent a boost from 0.66 and 0.48 to 0.67 and 0.49, respectively, while there were no alterations in the efficiency scores of the other DMUs.

At this instant, another efficient DMU is to be considered while regarding DMU 22. In case we assume that the output vector of DMU 22 experiences a variation from (898, 1.31, 98) to (947, 1.30, 100) as well as assuming *W* ^*T*^ = (1, 1, 1, 1, 1, 1) for the input weights, no optimal solution was accordingly detected for DMU 22 after solving Model (3). To further elucidate, it needs to be asserted that this was the case since (947, 1.30, 100) did not appear in *P*_out_. Again, we need to take into account DMU 22 while there is a variation in the output vector of DMU 5 from (898, 1.31, 98) to (947, 1.29, 99). This time, an optimal solution to Model (3) for DMU 22 is found and the reason behind this is that the new outputs for DMU 22 stay in *P*_out_. In further detail, the optimal solution would be Δ*x*_1_ = 0.09, Δ*x*_2_ = −0.89, Δ*x*_3_ = −1.36, Δ*x*_4_ = 37.50, Δ*x*_5_ = 5, Δ*x*_6_ = 6.90 while the optimal objective value would be 366.24. It can be then observed that the new input vector would be (21.09, 6.11, 74.64, 127.50, 10, 126.90). At last, it needs to be asserted that all the DMUs efficiency scores can be estimated by Model (5) while utilizing the new input and output vectors for DMU 22 for the remainder of the DMUs. Moreover, some improvement is discerned in the efficiency scores of DMU 15 and 23, changing from 0.78 and 0.45 to 0.81 and 0.46, respectively. Contrariwise, no change was observed in the efficiency scores of the rest of the DMUs.

## 6. Concluding Remarks and Further Research

If it is intended to specify the optimum potential values for the inputs (outputs) of any output values (inputs) in a deliberated DMU, the traditional inverse DEA model will be commonly utilized so that the relative efficiency value of the deliberated DMU would be steady in comparison with the other DMUs. In the current paper, a new invers DEA model was introduced which revolves around directional slack-based measures of efficiency with general returns to scale assumption of technology. In fact, the efficiency analyses of the entire DMUs are considered by the proposed inverse model which is unlike the inverse DEA models proposed by other surveys. In further detail, the inverse proposed model can be utilized with the aim of determining the optimum potential input values for certain outputs values of an efficient DMU. In this way, it is emphasized that the efficiency score associated with every DMU does not worsen in a new production possibility set. To be precise, the efficiency index associated with every DMU will be retained while having a possibility of improvement, as well. The considered efficient DMU was eliminated in this method from the present PPS while being substituted by the same efficient DMU following the alterations implemented to its inputs and outputs. Furthermore, the proposed inverse model for the resource allocation problem was examined in this paper in which it was possible to consider the upsurges (decreases) of certain outputs concurrently. Another point to underscore is that the proposed approach is advantaged by its simplicity while it can be simply extended in diverse directions. For example, it is possible to employ the proposed models for discretionary and nondiscretionary data or the fuzzy data.

## Figures and Tables

**Table 1:** The Data and Results Obtained from Model (3) with One-Input and One-Output.

DMU	Existing		Efficiency
	*x*	*y*	ψ*
A	4	8	1.00
B	7	11	1.00
C	6	8	0.67
D	3	3	1.00
E	8	9	0.56
F	5	10	1.00
G	5	5	0.68
H	3.6	6	1.00

**Table 2:** The Data and Results Obtained from Model (3) with Two-Input and Two-Output.

DMU	Existing				Efficiency
	*x*_1_	*x*_2_	*y*_1_	*y*_2_	ψ*
1	10	20	70	6	1.00
2	15	15	100	3	1.00
3	20	30	80	5	0.45
4	25	15	100	2	0.86
5	12	9	90	8	1.00

**Table 3:** The Data and Results Obtained from Model (3) for the Die Press Separation of the Motorcycle-Part Company.

DMU	Existing									Efficiency
	*x*_1_	*x*_2_	*x*_3_	*x*_4_	*x*_5_	*x*_6_	*y*_1_	*y*_2_	*y*_3_	ψ*
1	18	8	93	140	5	110	861	1.27	98	1.00
2	18	7	87	120	5	80	947	1.27	96	1.00
3	18	7	83	110	5	70	906	1.29	96	1.00
4	24	14	163	260	15	210	672	1.27	96	0.47
5	21	5	62	120	5	90	974	1.27	98	1.00
6	21	7	79	150	20	110	867	1.28	100	1.00
7	18	9	102	210	20	150	806	1.27	98	1.00
8	18	8	95	110	20	80	869	1.29	97	1.00
9	24	14	136	320	15	240	696	1.28	97	0.47
10	24	9	98	220	20	170	817	1.28	100	0.80
11	24	8	94	110	15	120	913	1.23	100	1.00
12	24	6	82	170	15	120	989	1.27	99	1.00
13	18	8	97	130	15	170	823	1.29	95	1.00
14	18	7	89	90	15	130	862	1.32	97	1.00
15	21	7	79	180	15	140	888	1.31	98	0.78
16	21	7	87	110	10	130	847	1.34	97	1.00
17	21	9	113	140	10	80	793	1.27	98	1.00
18	21	6	74	130	10	130	957	1.29	99	1.00
19	21	7	79	110	10	110	842	1.32	99	1.00
20	21	8	95	200	10	170	839	1.28	97	0.66
21	18	7	74	170	15	110	887	1.26	98	1.00
22	21	7	76	90	5	120	898	1.31	98	1.00
23	24	16	187	290	15	280	614	1.29	98	0.45
24	24	13	175	210	15	260	735	1.28	95	0.48
25	18	8	91	130	20	120	885	1.31	96	1.00
